# Sestrin prevents atrophy of disused and aging muscles by integrating anabolic and catabolic signals

**DOI:** 10.1038/s41467-019-13832-9

**Published:** 2020-01-13

**Authors:** Jessica Segalés, Eusebio Perdiguero, Antonio L. Serrano, Pedro Sousa-Victor, Laura Ortet, Mercè Jardí, Andrei V. Budanov, Laura Garcia-Prat, Marco Sandri, David M. Thomson, Michael Karin, Jun Hee Lee, Pura Muñoz-Cánoves

**Affiliations:** 1 Department of Experimental & Health Sciences, University Pompeu Fabra, CIBERNED, 08003 Barcelona, Spain; 2 Centro Nacional de Investigaciones Cardiovasculares, 28019 Madrid, Spain; 3 School of Biochemistry and Immunology, Trinity Biomedical Sciences Institute, Trinity College Dublin, Dublin, D02 R590 Ireland; 4 Engelhardt Institute of Molecular Biology, Center for Precision Genome Editing and Genetic Technologies for Biomedicine, 119991 Moscow, Russia; 5 Department of Biomedical Science, University of Padova, 35100 Padova, Italy; 6 Department of Physiology and Developmental Biology, Brigham Young University, Provo, UT 84602 USA; 7 Department of Pharmacology, University of California San Diego, La Jolla, CA 92093 USA; 8 Department of Molecular and Integrative Physiology, University of Michigan, Ann Arbor, MI 48109-2200 USA; 9 ICREA, 08003 Barcelona, Spain; 10 Present Address: Instituto de Medicina Molecular (iMM), Faculdade de Medicina, Universidade de Lisboa, 1649 Lisbon, Portugal; 11 Present Address: Princess Margaret Cancer Centre, University Health Network, Toronto, M5G 1L7 ON Canada

**Keywords:** Autophagy, Proteolysis, Skeletal muscle

## Abstract

A unique property of skeletal muscle is its ability to adapt its mass to changes in activity. Inactivity, as in disuse or aging, causes atrophy, the loss of muscle mass and strength, leading to physical incapacity and poor quality of life. Here, through a combination of transcriptomics and transgenesis, we identify sestrins, a family of stress-inducible metabolic regulators, as protective factors against muscle wasting. Sestrin expression decreases during inactivity and its genetic deficiency exacerbates muscle wasting; conversely, sestrin overexpression suffices to prevent atrophy. This protection occurs through mTORC1 inhibition, which upregulates autophagy, and AKT activation, which in turn inhibits FoxO-regulated ubiquitin–proteasome-mediated proteolysis. This study reveals sestrin as a central integrator of anabolic and degradative pathways preventing muscle wasting. Since sestrin also protected muscles against aging-induced atrophy, our findings have implications for sarcopenia.

## Abstract (web summary)

Ageing is associated with muscle atrophy, which negatively impacts quality of life. Here the authors show that expression of sestrins decreases during inactivity and that their overexpression prevents atrophy in mice via modulation of autophagy and protein degradation.

## Introduction

The control of mass in adult skeletal muscle is determined by a dynamic balance between anabolic and catabolic processes triggered by changes in activity or pathological conditions^1^. Muscle hypertrophy is associated with increased protein synthesis induced by activated AKT and mammalian target of rapamycin complex 1 (mTORC1) pathways^1^. Unlike muscle hypertrophy, muscle atrophy always involves a proteostatic shift in favor of catabolic versus anabolic processes^1,2^. Chief among these implicated catabolic changes is the activation of the autophagy-lysosome and ubiquitin–proteasome pathways, in particular the induction of muscle-specific ubiquitin ligases of the atrophy-related gene family, also known as atrogenes. Atrogenes, which are regulated by FoxO transcription factors (TF), remove proteins and organelles in atrophying fibers, whereas anabolic myofiber growth depends on AKT and mTOR activation^3–7^. AKT phosphorylates FoxO TF and impedes their nuclear activity, thus suppressing FoxO-dependent atrogene expression^8^. A complex scenario is thus emerging in which catabolic signaling connects with biosynthetic pathways during muscle atrophy, although little is known about how these connections are established.

Skeletal muscle atrophy is a major health problem and is the consequence of a wide variety of pathological conditions, including inactivity (immobilization or nerve injury), chronic diseases, and neuromuscular disorders. Muscle atrophy also complicates many aging-associated diseases, lowers life quality, and increases mortality. Regardless of the driving origin, muscle atrophy always involves loss of muscle mass, strength, and function^1,2^. Preventing or reversing muscle atrophy is therefore of the utmost importance, yet the mechanisms driving muscle atrophy are largely unknown.

Through a transcriptomic/bioinformatic screen for potential atrophy regulators, we identify the sestrin genes, particularly sestrin1 (but also sestrin 2), as genes downregulated rapidly in several models of muscle atrophy in vivo, including disuse, denervation, and aging (sarcopenia). Sestrins are a family of stress-inducible metabolic regulators that are conserved throughout metazoans^9^. Cell-based studies showed that sestrins have an antioxidant function that suppresses reactive oxygen species (ROS)^9,10^. Genetic studies of *Drosophila* sestrin (dSesn) revealed that, by activating AMPK, dSesn also functions as negative regulator of dTORC1, leading to several age-related pathologies^11^. Similar age-associated metabolic defects are also observed in cSesn-mutated *Caenorhabditis elegans*^12^. Recent studies indicate that mouse sestrins attenuate obesity-associated metabolic liver diseases, such as insulin resistance and steatohepatitis by suppressing oxidative stress or modulating AMPK/mTORC1 activity^10,13–15^. However, the role of mammalian sestrins in the regulation of muscle mass control is unknown, despite skeletal muscle being the site of maximal sestrin 1 (Sesn1) expression in humans and mice^16^.

In this study, we have made the surprising observation that not all catabolic activities are enhanced during muscle atrophy; rather, while proteasome activity is induced in response to inactivity, autophagy is blunted. Using sestrin gain-of-function and ablation approaches in mice, we find that sestrin preserves muscle mass and force in atrophying conditions by coordinating anabolic and catabolic pathways. This protective effect extends to age-induced muscle atrophy. Our results reveal that sestrin is a key instructor of skeletal muscle mass.

## Results

### Sestrins protect from disuse-induced muscle atrophy

**Fig. 1:**
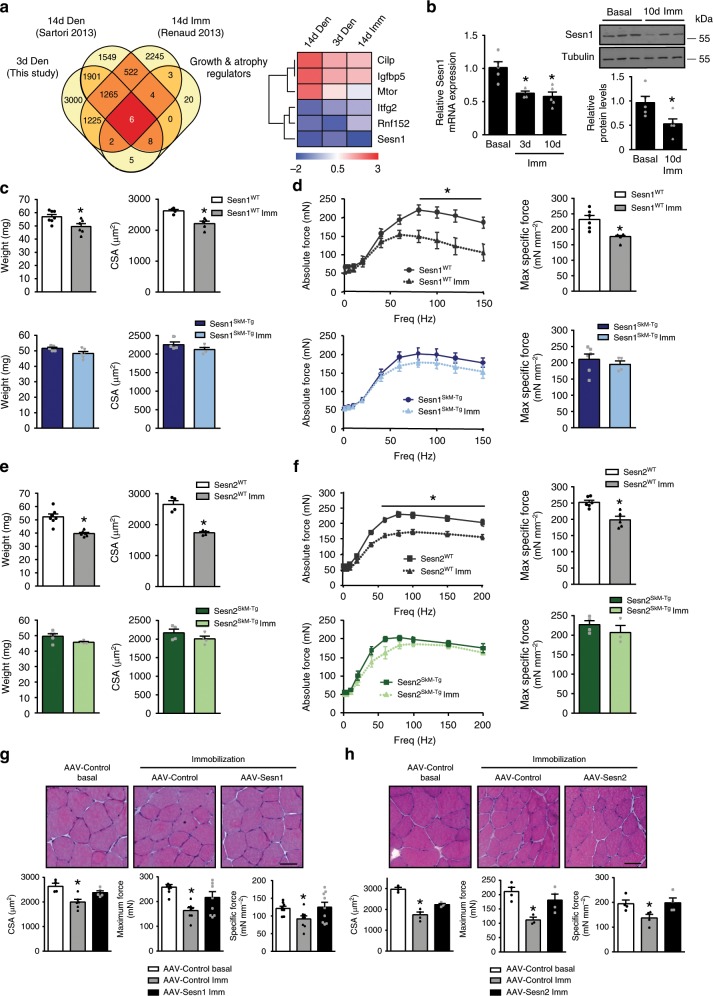
Sestrins prevent disuse-induced skeletal muscle atrophy. **a** *Left* Venn diagram showing overlap between a gene set of growth and atrophy regulators (see the “Methods” section) and dysregulated genes in immobilized (Imm) or denervated (Den) muscles reported in published muscle atrophy models or identified in our RNAseq comparison. *Right* Heat map for the six genes dysregulated in all gene sets analyzed. **b** Analysis of Sesn1 mRNA (left) and protein (right) in tibialis anterior (TA) muscles from non-immobilized (basal) and immobilized (Imm) limbs of WT mice for the indicated number of days (d). **c** TA muscle weight of and mean TA fiber cross-sectional area (CSA) in Sesn1^SkM-Tg^ mice (overexpressing human Sesn1) and corresponding wild type (WT) mice (Sesn1^WT^) in basal conditions and after 10 days of limb immobilization. **d** Force measurements in extensor digitorum longus (EDL) muscle of Sesn1^WT^ and Sesn1^SkM-Tg^ mice in basal conditions and after 10 days of limb immobilization. Charts show force–frequency curves (left) and maximum specific force (maximum force normalized by muscle area) (right). **e** Weight of TA muscles and mean TA fiber CSA in Sesn2^SkM-Tg^ mice (overexpressing human Sesn2) and corresponding WT mice (Sesn2^WT^) in basal conditions and after 10 days of limb immobilization. **f** Force measurements in EDL muscles of Sesn2^WT^ and Sesn2^SkM-Tg^ mice in basal conditions and after 10 days of limb immobilization. Charts show force–frequency curves and maximum specific force. **g** Histology and muscle force in EDL muscles of young mice transduced with AAV-Sesn1 or AAV-Control followed 4 days later by limb immobilization for 10 additional days. The upper panels show representative images of hematoxylin/eosin (H/E) staining in basal and immobilized muscles. Scale bar = 50 µm. The charts show fiber size (CSA), maximum force, and specific force. **h** Histology and muscle force in EDL muscles transduced with AAV-Sesn2 or AAV-Control and treated as described in **g**. Scale bar = 50 µm. All data are shown as mean with SEM. Statistical comparisons by unpaired two-tailed Student's *t*-test (*p < 0.05 vs. basal conditions). Sample numbers were *n* = 4–6 mice per group for **c**–**f** and *n* = 4 mice per condition for **g**, **h**. Source data are provided as a Source Data file.

Through a bioinformatic analysis of the atrophy-associated transcriptome, we searched for potential growth and atrophy regulators. Sesn1 was identified as one of six genes dysregulated in distinct models of muscle wasting in vivo, such as disuse and denervation (Fig. 1a and Supplementary Data 1). Sesn1 belongs to a conserved stress-inducible family of proteins with antioxidant and metabolic functions, which in vertebrates are encoded by the *Sesn1*, *Sesn2*, and *Sesn3* loci^9^. Sesn1, among sestrins, is highly expressed in mammalian skeletal muscle^16^ (Supplementary Fig. 1a). Considering that sestrins control mTOR and AKT pathways^9,11,14,17,18^, critical regulators of muscle function^1,2^, we examined the influence of Sesn1 downregulation on muscle atrophy.

Sesn1 RNA and protein expression decreased in mouse skeletal muscle after immobilization-induced limb inactivity (Fig. 1b), correlating with muscle atrophy and loss of force (Fig. 1c–f, white and gray charts). To investigate the involvement of Sesn1 in disuse atrophy, we generated transgenic mice overexpressing human Sesn1 (96% homologous to murine Sesn1) in skeletal muscle under the MCK promoter (Sesn1^SkM-Tg^ mice). Sesn1^SkM-Tg^ mice expressed Sesn1 in both fast and slow muscles, without changes in Sesn2 and Sesn3 expression (Supplementary Fig. 1b, c), were of normal weight, and showed no obvious muscle abnormalities under normal conditions when compared to littermate Sesn1^WT^ (wild-type, WT) mice (Supplementary Fig. 1d, e). However, Sesn1^SkM-Tg^ mouse muscle, but not the control Sesn1^WT^ muscle, was strongly protected against all measures of disuse atrophy, including muscle weight, myofiber size, fibrosis index, and force production by the extensor digitorum longus (EDL) (Fig. 1c, d, navy and blue charts, and Supplementary Fig. 1f) and soleus muscles (Supplementary Fig. 1g, navy and blue charts). No changes in myonuclear number were observed (Supplementary Fig. 1h). Importantly, Sesn1 also protected from atrophy induced by denervation, as shown by the preservation of myofiber size and muscle force in Sesn1^SkM-Tg^ mice compared to Sesn1^WT^ mice upon sciatic denervation (Supplementary Fig. 1i, j).

Given the high sequence homology of mammalian sestrins (Sesn1–3), and to discriminate whether the atrophy-preventing effect was specific for Sesn1 or could also be exerted by another sestrin family member, we tested muscle atrophy protection with a transgenic mouse line overexpressing human Sesn2 (92% homologous to murine Sesn2) in skeletal muscle (Sesn2^SkM-Tg^ mice), in comparison to littermate control Sesn2^WT^ mice (Supplementary Fig. 2a–c). Sesn2^SkM-Tg^ overexpression provided similar protection from disuse atrophy and force loss (Fig. 1e, f and Supplementary Fig. 2d), indicating that, upon overexpression, Sesn1 and Sesn2 are equally effective at preventing immobilization-induced muscle atrophy.

Transgenic Sesn1/2 expression throughout development has the potential to produce adaptive effects unrelated to sestrin activity that may mask sestrin functions in the adult. To exclude this, we overexpressed Sesn1 and Sesn2 in skeletal muscle of 4-month-old WT mice via adeno-associated virus (AAV) transduction (Supplementary Fig. 2e, f). Consistent with the results from transgenic mice, AAV-based Sesn1 or Sesn2 overexpression in WT muscle also prevented disuse-induced muscle atrophy and weakness (Fig. 1g, h). Sestrins may function as antioxidants through their oxidoreductase activity^10,19^, prompting us to test the effect of an oxidoreductase-disrupting C130 mutation in Sesn^10,19^. Overexpression of Sesn1-C130S still impeded disuse muscle atrophy (Supplementary Fig. 2g), indicating that the protective action of sestrin is independent of its antioxidant function.

### Loss of Sesn 1 aggravates disuse-induced muscle atrophy

**Fig. 2:**
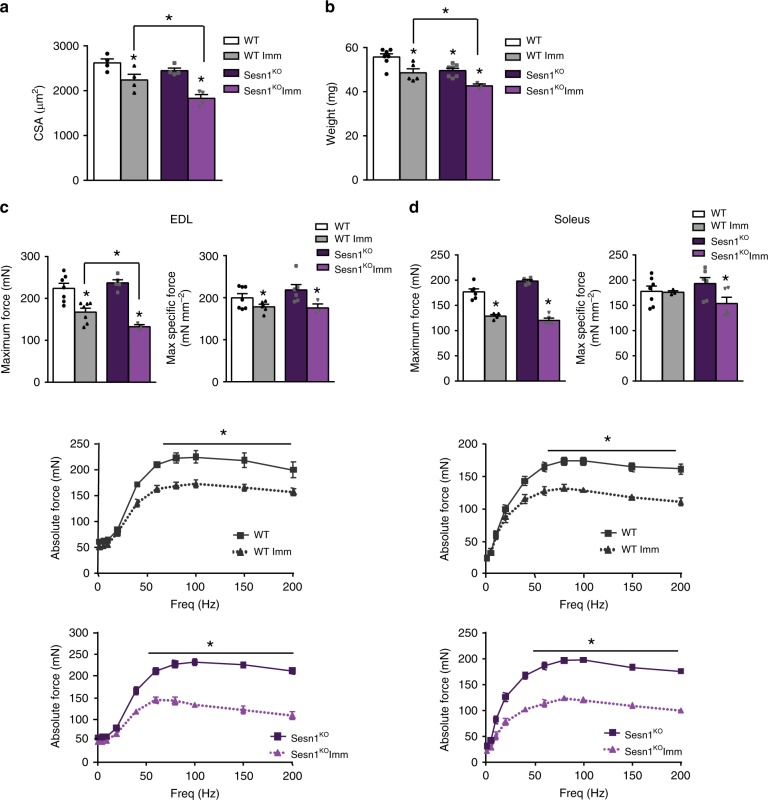
Loss of sestrin1 exacerbates disuse-induced muscle atrophy. **a** Mean CSA of TA fibers from WT and Sesn1^KO^ mice in basal conditions and after 10 days of limb immobilization. **b** TA muscle weight in WT and Sesn1^KO^ mice in basal conditions and after 10 days of limb immobilization. **c**, **d** Force measurements in EDL **c** and soleus **d** muscles of WT and Sesn1^KO^ mice in basal conditions and after 10 days of limb immobilization. Charts show maximum and specific force (top) and force–frequency curves (bottom). All data are shown as mean with SEM. Comparisons by unpaired two-tailed Student's *t*-test (*p < 0.05). *N* = 3–7 mice per genotype and condition. Source data are provided as a Source Data file.

Of the three sestrins, Sesn1 is the main form expressed in muscle^10,16,19^ (Supplementary Fig. 1a). We therefore analyzed mice deficient in Sesn1 (Sesn1^KO^ mice)^20^ in comparison to their WT controls. Although these mice showed no detectable alterations in muscle weight, myofiber size, or force under basal conditions (Supplementary Fig. 3a–d), they showed more pronounced myofiber atrophy and force loss in EDL and soleus muscles in response to inactivity (Fig. 2a–d). No changes in Sesn2/Sesn3 levels were found in muscles of Sesn1^KO^ mice (Supplementary Fig. 3e); Sesn1 is thus critical for preventing muscle wasting.

We next evaluated whether sestrin provided equal protection to distinct types of muscle fibers. Disuse muscle atrophy was associated with muscle fiber-type switching from IIA/IIX to IIB fibers in WT mice and in Sesn1^SkM-Tg^ mice (Supplementary Fig. 3f). Preservation of muscle mass upon Sesn1 overexpression and increased atrophy after Sesn1 deletion was observed in type IIA/IIX and type IIB fibers (Supplementary Fig. 3f, g), indicating that sestrin protects against muscle atrophy independently of fiber-type changes.

### Sestrin blunts FoxO-dependent atrogenes in disused muscle

**Fig. 3:**
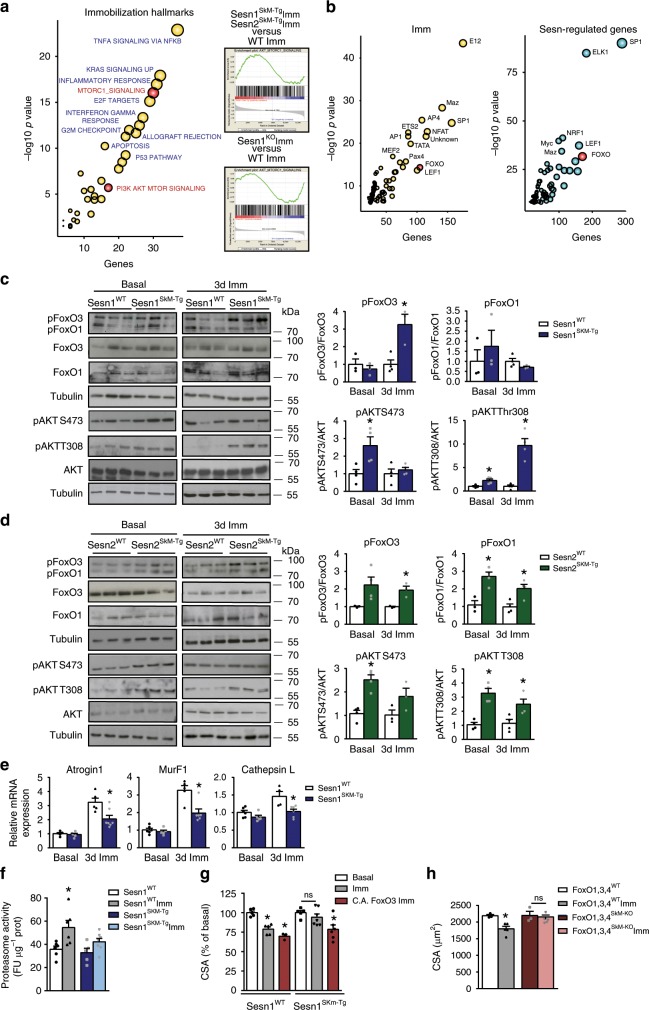
Sestrin blunts FoxO-dependent upregulation of muscle atrogenes. **a** Gene set enrichment analysis (GSEA) of immobilization-related genes. Bubble plot of enriched GSEA hallmarks in the dysregulated genes upon 3-day muscle immobilization in WT mice (left). Enrichment plots of the combined PI3K-AKT-MTOR signaling and mTORC1-signaling hallmarks (AKT-MTORC1 signaling) in comparisons of Sesn1/2^SkM-Tg^ vs. WT mice and Sesn1^KO^ vs. WT mice (right). **b** Bubble plot of enriched transcription factor-binding sites (GSEA) among immobilization-dysregulated genes (left) and the sestrin-regulated gene set defined in Supplementary Fig. 4a (right). **c** Western blot analysis showing phosphorylation levels of FoxO1, FoxO3, and AKT in muscles of Sesn1^WT^ and Sesn1^SkM-Tg^ mice in basal conditions and after 3 days of immobilization. Representative blots are shown (left) with corresponding quantification. For each experimental condition (basal and immobilization) values of Sesn1^SkM-Tg^ are referred to averaged values for Sesn1^WT^ samples, which were set to one. **d** Western blot analysis of the phosphorylation levels of FoxO1, FoxO3, and AKT in muscles of Sesn2^WT^ and Sesn2^SkM-Tg^ mice in basal conditions and after 3 days of immobilization. Representative blots are shown (left) with corresponding quantification (right), relatively to Sesn2^WT^ values, as in **c**. **e** Atrogin1, MurF1, and Cathepsin L mRNA levels in skeletal muscle from Sesn1^WT^ and Sesn1^SkM-Tg^ mice in basal conditions and after 3 days of immobilization. **f** Proteasome activity in total homogenates of TA muscles from Sesn1^WT^ and Sesn1^SkM-Tg^ mice in basal conditions and after 3 days of immobilization. **g** Mean myofiber CSA in TA muscle from Sesn1^WT^ and Sesn1^SkM-Tg^ mice electrotransferred with control vector or with a plasmid encoding constitutively active FoxO3 (C.A. FoxO3) and then immobilized for 10 days. Values are relative to basal conditions. **h** Mean myofiber CSA in TA muscle from FoxO1,3,4^WT^ and FoxO1,3,4^SkM-KO^ mice in basal conditions and after 10 days of immobilization. All data are shown as mean with SEM. Comparisons by Student's *t*-test (*p < 0.05). Sample numbers were *n* = 3–4 mice per group for **c**, **d**, *n* = 3–6 animals for **e**–**g** and *n* = 3–5 mice per genotype and condition for **h**. Source data are provided as a Source Data file.

To interrogate the mechanistic basis of sestrin-mediated protection against muscle atrophy, we performed RNAseq analysis on control and immobilized TA muscles of all available mouse genotypes: Sesn1^SkM-Tg^, Sesn2^SkM-Tg^, and Sesn1^KO^ mice, and their respective WT counterparts. Like other atrophy models^21^, muscle immobilization enriched gene expression signatures for apoptosis, inflammation, cell-cycle inhibition, and anabolic regulation in all mouse groups (Fig. 3a and Supplementary Data 2). Interestingly, the association between immobilization and anabolic signaling hallmarks (PI3K/AKT/mTORC1 regulation) was strengthened by Sesn1/2 overexpression, whereas these hallmarks correlated negatively with Sesn1 deficiency (Fig. 3a).

We also performed a hierarchical clustering analysis to identify genes regulated by both immobilization and sestrins (Supplementary Fig. 4a). We focused on gene clusters whose immobilization-dependent induction was increased or decreased by Sesn overexpression (Clusters A and B) but were behaving contrarily in Sesn1-deficient muscles (Clusters C and D) (Supplementary Fig. 4a and Supplementary Data 3). Intersection between clusters A/C and clusters B/D yielded a gene set (defined as Sestrin-regulated genes) that was highly enriched in canonical pathways implicated in ubiquitin–proteasome-mediated proteolysis, including proteasome subunits and the E3 ubiquitin-ligase atrogenes MuRF1 (*Trim63*) and Atrogin1 (*Fbxo32*)^6,22–24^ (Supplementary Fig. 4a and Supplementary Data 3). Other proteostasis pathways, such as macroautophagy-lysosome degradation, were also enriched (Supplementary Fig. 4b). Interestingly, genes regulated by immobilization and sestrins were often found to contain binding sites for FoxO, Myc/Maz, and Sp1 TF (Fig. 3b). FoxO TF-binding sites were highly enriched in atrogenes and autophagy genes present in the sestrin-regulated gene set (Supplementary Fig. 4c, left and right). Of note, a significant overlap was found between sestrin-regulated atrogenes and a previously described list of FoxO-regulated atrogenes^24^ (Supplementary Fig. 4c, middle). These results are consistent with the former findings that upregulation of atrogenes, particularly *Atrogin1* and *MuRF1*, is directed by FoxO3a and required for muscle atrophy^3,6,7,24^.

Based on these findings, we examined the status of AKT-FoxO signaling in immobilized muscle of Sesn1/2 transgenic mice. Immobilized muscles of Sesn1^SkM-Tg^ and Sesn2^SkM-Tg^ mice had higher levels of AKT and FoxO3 phosphorylation than WT counterparts (Fig. 3c, d; Supplementary Fig. 5a). Notably, sestrin overexpression in muscle upregulated both PDK1-dependent AKT phosphorylation at Thr308 and mTORC2-dependent phosphorylation at Ser473 (Fig. 3d). Phosphorylation at both sites activates AKT kinase activity, while AKT-mediated phosphorylation of FoxO inhibits its transcriptional activity by retaining it in the cytoplasm^8^. Consequently, immobilized Sesn1/2^SkM-Tg^ muscle exhibited lower expression of well-known FoxO targets^24^ such as *Atrogin1* and *MuRF1* (Fig. 3e and Supplementary Fig. 5b), consistent with reduced FoxO transcriptional activity and with the transcriptome studies (Supplementary Figs. 4b, c and  5c). In contrast, disused muscle of Sesn1^KO^ mice expressed higher levels of these atrogenes (Supplementary Fig. 5d). In line with atrogene-mediated upregulation of ubiquitin–proteasome activity, the immobilization-induced increase in chymotrypsin-like proteasome activity in muscles of WT mice was blunted by sestrin overexpression (Fig. 3f). Conversely, blocking proteasome activity with bortezomib prevented the disuse atrophy in WT mice (Supplementary Fig. 5e). These results suggest that sestrins protect disused muscles from wasting, at least in part, by repressing the induction of FoxO-regulated atrogenes encoding muscle proteolytic enzymes.

Involvement of FoxO in sestrin-mediated muscle protection was confirmed through genetic modulation of FoxO. Muscle-specific overexpression of constitutively active FoxO3 (FoxO3 TM^7^) abolished the protective effect of Sesn1 against muscle atrophy in Sesn1^SkM-Tg^ mice (Fig. 3g). Contrarily, muscle-specific genetic deletion of all three FoxO genes (FoxO1,3,4^SkM-KO^ triple-knockout mice) (Supplementary Fig. 5f) produced strong protection against inactivity-induced muscle atrophy-like Sesn1/2 overexpression (Fig. 3h, see Fig. 1 for comparison). AKT activation by sestrin thus blocks ubiquitin–proteasome-dependent proteolysis via FoxO-signaling inhibition, subsequently attenuating muscle wasting during disuse.

### Sestrin coaxes autophagy by blunting mTORC1 to protect muscle

**Fig. 4:**
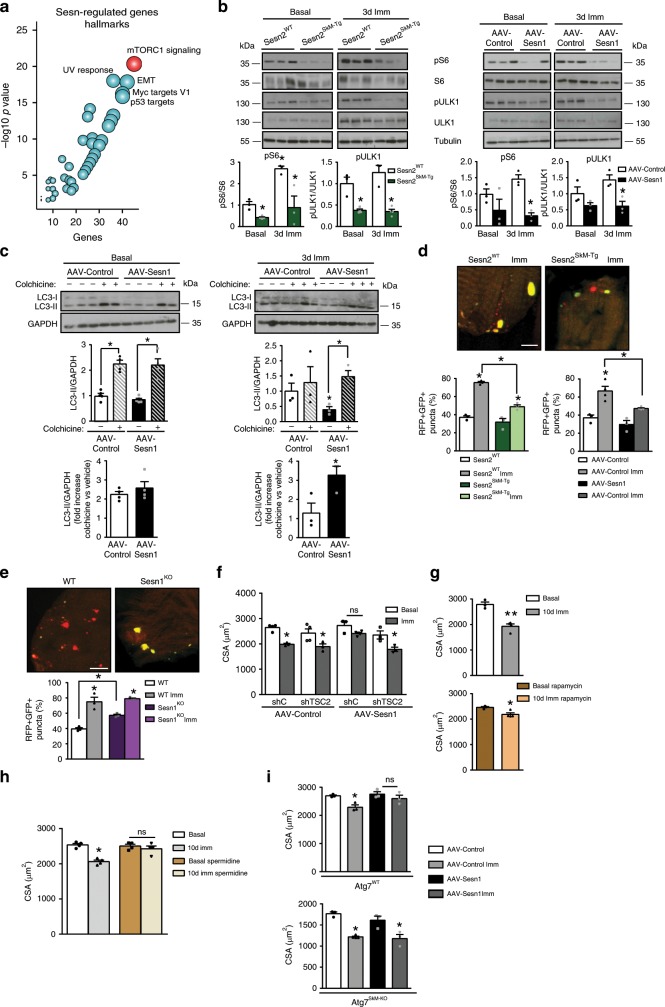
Autophagy induction via sestrin-mediated mTORC1 blockade prevents atrophy. **a** Bubble plot showing the main molecular hallmarks (GSEA) enriched in the sestrin-regulated gene set defined in Supplementary Fig. 4a. **b** Western blot analysis and quantification of S6 and ULK1 phosphorylation in muscles from Sesn2^WT^ and Sesn2^SkM-Tg^ mice (left) and in muscles transduced with AAV-Sesn1 or AAV-Control (right) in basal conditions and at 3 days post-immobilization. Values were normalized to basal control conditions. **c** Western blot analysis and quantification of LC3I and LC3II in TA muscles transduced with AAV-Sesn1 or AAV-Control in basal conditions and at 3 days post-immobilization. Treatment of mice with colchicine or vehicle is indicated. Lower chart shows the fold increase in LC3II content in colchicine-treated versus vehicle-treated mice. **d** Representative confocal images of 3-day-immobilized TA muscle electrotransferred with a tandem mRFP-GFP-LC3 reporter plasmid to enable detection of autophagosomes (yellow puncta) and autolysosomes (red puncta). The chart shows double RFP^+^GFP^+^ puncta as a percentage of total puncta for the indicated genotypes. Scale bar = 5 µm. **e** Representative confocal images of non-immobilized muscles in WT and Sesn1^KO^ mice treated as in **d**. The chart shows RFP+GFP+ puncta as a percentage of total puncta. Scale bar = 5 µm. **f** Mean TA myofiber CSA in basal conditions and at 10 days post-immobilization in muscles transduced with AAV-Control or AAV-Sesn1 and electrotransferred with control short hairpin (sh) or sh targeting TSC2. **g** Mean TA myofiber CSA in basal conditions and at 10 days post-immobilization in WT mice treated with vehicle (top) or rapamycin (bottom). **h** Mean TA myofiber CSA in basal conditions and at 10 days post-immobilization in WT mice treated with vehicle or spermidine. **i** Mean TA myofiber CSA in basal conditions and at 10 days post-immobilization in Atg7^WT^ and Atg7^SkM-KO^ mice transduced with AAV-Control or AAV-hSesn1. All data are shown as mean with SEM. Comparisons by Student's *t*-test (*p < 0.05 and **p < 0.01 vs. basal). Sample numbers were *n* = 3 mice per group for **b**, **d**, **e**, *n* = 3–5 animals for **c**, **f**, **g**, **i** and *n* = 4 for **h**. Source data are provided as a Source Data file.

Gene set enrichment analysis (GSEA) also identified mTORC1-signaling regulation as another hallmark of sestrin modulation in muscle (Fig. 4a). mTORC1 is a major anabolic factor, classically associated with promotion of muscle growth and hypertrophy. Although mTORC1 was originally thought to decline during muscle atrophy^1,2^, we found sustained—and even increased—mTORC1 activity in immobilized atrophic muscles of non-transgenic mice, as revealed by phosphorylation of the mTORC1 downstream targets S6 and ULK (Fig. 4b). Overexpression of Sesn1 or Sesn2, which restores muscle mass in the disuse condition, strongly inhibited mTORC1 signaling (i.e. reduced S6 and ULK1 phosphorylation) after immobilization (Fig. 4b), whereas Sesn1^KO^ muscle showed constitutive mTORC1-signaling activation even in resting muscle (Supplementary Fig. 6a). Therefore, muscle immobilization unexpectedly conveys mTORC1 activation, whereas sestrins strongly downregulate it.

mTORC1-mediated ULK1 phosphorylation inhibits autophagy^13,25^, another major protein and organelle degradation pathway. The autophagy pathway was also found in the sestrin-regulated gene set, which included many autophagy-related genes enriched in FoxO TF-binding sites (Supplementary Fig. 4b, c and Supplementary Data 3). Skeletal muscle autophagy flux was determined in vivo using colchicine, which blocks autophagosome degradation^26^. Colchicine-triggered marked accumulation of the autophagosome marker LC3-II in WT and sestrin-overexpressing muscles (Fig. 4c, left panel, and Supplementary Fig. 6b), indicating that basal autophagy flux is active in all these tissues, being barely affected by Sesn1/2 overexpression in non-disuse conditions. However, muscle immobilization in WT mice strongly decreased colchicine-dependent LC3-II accumulation (Fig. 4c, right panel, and Supplementary Fig. 6c), indicating that autophagy flux was attenuated. Importantly, autophagy flux was preserved in immobilized muscles overexpressing Sesn1 or Sesn2 (Fig. 4c, right panel, and Supplementary Fig. 6c). This observation is consistent with downregulation of mTORC1-dependent ULK1 activation by overexpression of sestrins during immobilization conditions (Fig. 4b). These findings were reinforced by experiments with a tandem fluorescent autophagy flux reporter (mRFP-GFP-LC3^27^) (Fig. 4d). Although mature autolysosomes (red LC3 puncta) were abundant in basal WT muscle, the red LC3 signal was eliminated by muscle immobilization, leaving only yellow puncta (undegraded autophagosomes). Sesn1/2 overexpression substantially preserved the red LC3 puncta during immobilization (Fig. 4d). Conversely, Sesn1^KO^ mice showed impaired autophagy flux already in basal conditions, with reduced colchicine-dependent LC3-II accumulation (Supplementary Fig. 6d) and loss of red LC3 puncta in the reporter assay (Fig. 4e). The defective autophagy in non-immobilized muscle of Sesn1^KO^ mice could be explained by the constitutive mTORC1-dependent ULK1 activation in the absence of sestrin (Supplementary Fig. 6a), since stress-induced sestrin expression inhibits mTORC1 signaling via activation of AMPK^28^. Consistent with this idea, AMPK activation was readily observed in non-immobilized Sesn1-overexpressing muscle, providing a mechanistic explanation for how sestrin inhibits mTORC1 and activates autophagy (Supplementary Fig. 6e).

We further tested whether sestrin-induced autophagy is important for attenuating disuse atrophy through genetic and pharmacological approaches. Sesn1-mediated protection against disuse atrophy was abolished upon constitutive mTORC1 activation by silencing TSC2, the negative regulator of mTORC1^29–31^ (Fig. 4f and Supplementary Fig. 6f). Conversely, rapamycin, which inhibits mTORC1 and induces autophagy^32^, increased WT muscle autophagy flux (Supplementary Fig. 7a), and substantially attenuated muscle atrophy (Fig. 4g), like sestrins. A similar protective effect against muscle atrophy was exerted by the autophagy inducer spermidine^32,33^ (Fig. 4h). Autophagy activation through Atg7 overexpression^32^ also protected myofibers from disuse-induced atrophy (Supplementary Fig. 7b, c), whereas muscle-specific genetic deletion of Atg7 (Atg7^SkM-KO^) (Supplementary Fig. 7d) nullified Sesn1-mediated protection against disuse-induced muscle atrophy (Fig. 4i). No significant crosstalk between both catabolic activities was observed during muscle immobilization (not shown). Taken together, these data demonstrate that muscle inactivity produces detrimental effects by differentially acting on catabolic mechanisms (i.e. decreasing autophagic while inducing proteasomal pathways). By reversing this concert, sestrins preserve autophagy that is essential for muscle homeostasis, while preventing proteasome overactivation that wastes muscle by majorly driving loss of muscle mass during disuse condition.

### Sestrins protect muscle against aging-associated atrophy

**Fig. 5:**
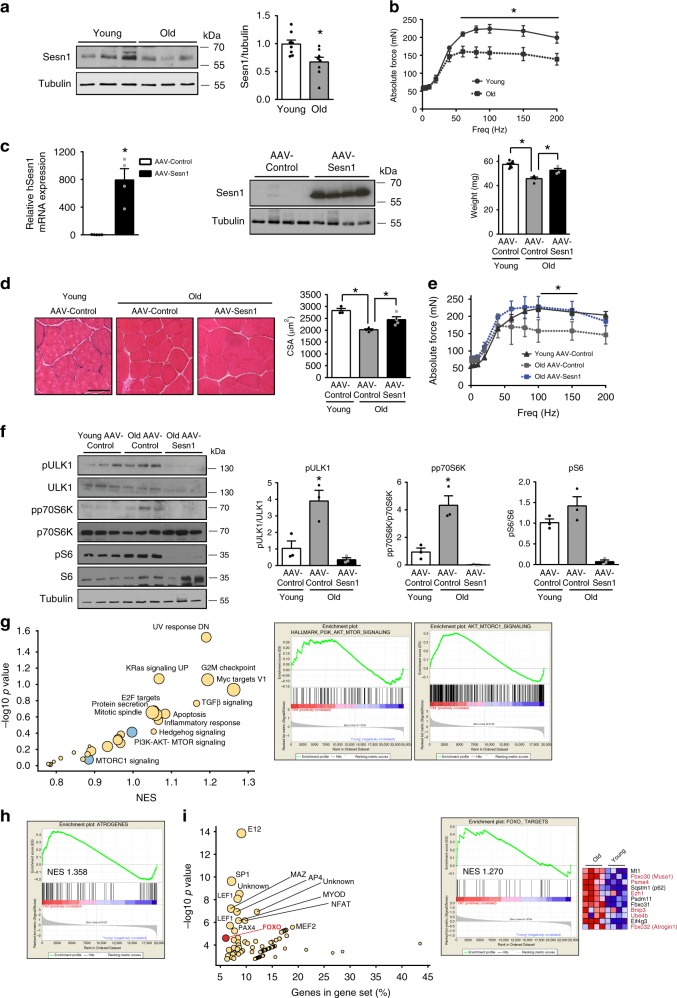
Sestrins prevent aging-related muscle atrophy. **a** Western blot and quantification of Sesn1 protein levels in skeletal muscle from young (4 months) and old (24 months) mice. **b** Force–frequency curve of EDL muscles from young and old WT mice. **c** qPCR of human Sesn1 mRNA in skeletal muscle from 24-month-old mice transduced with AAV-Sesn1 for one month and Sesn1 protein expression by Western blotting in the same muscles (left). Weight of TA muscles from young and old mice transduced with AAV-Sesn1 or AAV-Control for 1 month (right). **d** Representative H/E staining pictures of TA muscle sections from 24-month-old mice transduced with AAV-Sesn1 or AAV-Control for 1 month and quantification of mean myofiber CSA. Scale bar = 50 µm. **e** EDL force-frequency curve in 24-month-old mice transduced with AAV-Sesn1 or AAV-Control for 1 month. **f** Western blot analysis and quantification of phosphorylation levels of p70S6K, S6, and ULK1 in muscles from old mice transduced with AAV-Sesn1 or AAV-Control for 1 month. Representative blots are shown with corresponding quantification. Values were normalized to averaged values of young control mice. **g** Bubble plot of enriched GSEA hallmarks in the dysregulated genes upon muscle aging (left). Enrichment plots of the PI3K-AKT-MTOR signaling hallmark and the combined PI3K-AKT-MTOR signaling and mTORC1 signaling hallmarks (AKT-MTORC1 signaling) in comparisons of old and young mice (right). **h** As in **g**, enrichment plots of the atrogenes gene set in comparisons of old and young mice. **i** Bubble plot of enriched transcription factor-binding sites (GSEA) in the dysregulated genes upon muscle aging (left). Enrichment plots of the FoxO3a targets in skeletal muscle (as defined in Brocca et al.^24^) and heatmap illustrating genes with higher enrichment in comparisons of old and young mice (right). All data are shown as mean with SEM. Comparisons by Student's *t*-test (*p < 0.05). Sample numbers were *n* = 8 mice per group for **a**, *n* = 4–7 mice per condition for **b** and **c**, *n* = 3–5 mice per condition for **d** and **e** and *n* = 3 mice per group for **f**. Source data are provided as a Source Data file.

We finally investigated whether sestrins can also protect against aging-associated muscle atrophy (sarcopenia). Compared with young mice (4 months old), aged mice (24 months old) showed lower expression of Sesn1 protein in skeletal muscle (Fig. 5a) accompanied by pronounced loss of skeletal muscle force and mass and myofiber atrophy (Fig. 5b–e). All these muscle parameters were improved by transduction of AAV-Sesn1 for one month, including a slight reduction in muscle fibrosis (Fig. 5c–e and Supplementary Fig. 7e), suggesting that sestrin activation presents a promising strategy for protecting against age-associated muscle atrophy and related conditions. No changes in myonuclear number were observed (Supplementary Fig. 7f). Mechanistically, we found that mTORC1 activity is increased in muscles of aged mice and is reduced by Sestrin overexpression (Fig. 5f), correlating with the protection exerted by Sestrin on age-related muscle atrophy.

Finally, we compared publicly available transcriptomic data of skeletal muscles of young and old mice (GEO: GSE53959)^34^ to assess for potential gene expression similarities between aged and immobilized muscles. Interestingly, we found that aged-muscle transcriptome was enriched in gene expression signatures for apoptosis, inflammation, cell-cycle inhibition, UV response, Myc targets, and anabolic signaling (PI3K/AKT/mTORC1 regulation) (Fig. 5g). Supporting the existence of commonalities between genes induced by muscle immobilization and aging, the aged muscle transcriptome was found to be also enriched in atrogenes (Fig. 5h), while the upregulated genes contained binding sites for E12, Myc/Maz, Sp1, and FoxO TFs (Fig. 5i). In fact, the transcriptome of old mice was enriched in FoxO and Sestrin-regulated atrogenes^24^, including the E3 ubiquitin-ligases *Atrogin1* and *Musa1* (*Fbxo30*)^23^ and autophagy-related genes *Bnip3* and p62 (*Sqstm1*) (Fig. 5i). Taken together, these results suggest that, similar to disuse-induce muscle atrophy, sestrins may protect muscle against aging-associated muscle atrophy by coordinating anabolic and catabolic pathways.

## Discussion

Autophagy and ubiquitin–proteasome proteolytic activities have been independently linked to muscle wasting^6,31,35^. Our analysis combining unbiased transcriptomics and targeted transgenesis approaches has identified an important mechanism for protecting muscle from wasting during disuse and aging. Sestrins, strongly downregulated by disuse, preserve muscle mass by coordinately inhibiting atrophic proteolysis and activating homeostatic autophagy. Sestrins may achieve disuse-induced protection by regulating the balance between distinct growth-associated mTOR complexes, strongly inhibiting mTORC1^28^ while supporting mTORC2 activity^14^. Through upregulation of AKT, sestrins inhibit FoxO-dependent transcription of atrogenes, which normally promote muscle wasting by accelerating protein degradation. At the same time, through the activation of AMPK and inhibition of mTORC1^28^, sestrins upregulate autophagy, thus maintaining proteostasis and organelle quality for homeostatic preservation of muscle mass and force. Of interest, the protective effect of sestrin was also extended to denervation-induced muscle wasting and age-associated muscle atrophy. The relevance of our data is further supported by recent findings demonstrating reduction of sestrins’ protein levels in muscles of aging human individuals^36^ including muscles from the frail elderly population^37^. Future studies should explore the effect of Sestrin/mTOR modulators such as NV-5138^38^ on these conditions. Loss of muscle mass and force significantly affects health, life quality, and even survival. Given their beneficial effects on muscle wasting during disuse and aging, sestrins should be regarded as nodal regulators of mammalian muscle growth with potentially broad applications in the treatment of common catabolic conditions.

## Methods

### Animals

The different mouse models used in this study were generated as follows:

The skeletal muscle-specific Sesn1 transgenic mouse model (Sesn1^SkM-Tg^) was generated in C57BL/6 background and carry a transgene coding for human Sesn1 under the control of MCK promoter. The human Sesn1 cDNA sequence (from LV-Sesn1^19^) was subcloned into the pBluescript-MCK plasmid (a kind gift from Markus Rüegg). A 4 kb PacI digestion fragment was excised, microdialyzed, and microinjected into the pronuclei of fertilized mouse eggs (C57BL/6J × C57BL/6J) at the Mouse Mutant Core Facility, Institute for Research in Biomedicine (Barcelona, Spain). Embryos were implanted into pseudo-pregnant foster females (ICR), and transgenic pups were identified. DNA samples from tail clips of subsequent litters were screened by PCR with primers spanning different sequences of MCK promoter and Sesn1 cDNA (Primer set 1 forward CTGCCCCCGGGTCACCACC reverse TCGATTCAGGTCATATAGCGGGT; Primer set 2 forward CAGGGCTTATACGTGCCTGGGACTC reverse TGTGGGTGGAAAACCATCACTAACG; Primer set 3 forward GCCCCCGGGTCACCACCAAG reverse GGCCAAGCGCATGGATCCTTTTA) that amplified a 1550, 600, and 76 bp fragments, respectively. The transgene was maintained on the C57BL/6J background throughout the study.

The skeletal muscle-specific Sesn2 transgenic mouse model (Sesn2^SkM-Tg^) was generated by crossing mice that express Sesn2 from a Tet-regulated promoter (provided by Dr. M. Karin, UCSD, San Diego, USA) with mice harboring the skeletal muscle-specific MCK-tTA construct (obtained from Dr. M. Ruegg, Biozentrum, Basel, Switzerland). Non-transgenic littermates (that were WT for Sesn1 and Sesn2 expressions) were used as controls for the two transgenic mouse lines, and named Sesn1^WT^ and Sesn2^WT^ mice, respectively. Sesn1 KO mice were provided by Dr. J.H. Lee (University of Michigan, USA) and analyzed in comparison with normal, control WT mice. Atg7^SkM-KO^ mice were generated as previously described^32^, and FoxO1,3,4^SkM-KO^ mice were obtained by crossing FoxO1/3/4-floxed mice (a kind gift from Dr. M. Sandri, University of Padova, Italy) with the *Pax7*^Cre^ line (provided by Dr. M. Capecchi, University of Utah, USA). Non-transgenic littermates (that were WT for FoxO1,3,4 and for Atg7 expression) were used as controls for Atg7^SkM-KO^ mice and for FoxO1,3,4^SkM-KO^ mice, and named Atg7^WT^ and FoxO1,3,4^WT^ mice, respectively. Therefore, Sesn1^WT^, Sesn2^WT^, WT, Atg7^WT^, and FoxO1,3,4^WT^ mice are all equally WT, non-transgenic mice, but each one is used as littermate control of each individual genetically modified mouse line.

Mice were housed in standard cages under 12 h light/dark cycles with ad libitum access to food and water. All experiments were performed on 4–5-month-old male mice. All animal experiments were approved by the Ethics Committee of the Barcelona Biomedical Research Park (PRBB) and performed according to Catalan and European legislation.

### Induction of muscle atrophy

The immobilization protocol was performed unilaterally on anesthetized animals as formerly described^39^. Briefly, the right hindlimb was immobilized with rigid plastic sticks fixed with a medical adhesive bandage. This procedure prevented movement of the immobilized leg alone. Denervation was performed as previously described^40^. In brief, a 5 mm segment of the sciatic nerve was surgically removed down to the gluteus maximum from the right leg. Mice not subjected to atrophy-promoting conditions were used as controls (Basal). Muscles were removed at 3 or 10 days after atrophy induction and frozen in liquid nitrogen for subsequent analyses.

### In vivo gene electrotransfer and AAV injection

Expression plasmids used for electrotransfer studies were purified using a Endofree plasmid kit (Qiagen) and dissolved in 0.9% NaCl. 45 min before electrotransfer, muscles were pretreated with hyaluronidase (10 U/muscle). Afterwards, naked plasmids (60 μg DNA) were injected into the tibialis anterior muscle and 10 pulses of 20 ms each were applied to each hindlimb at 175 V/cm and 1 Hz using an electroporator (ECM 830; BTX). Empty vector was used as control.

AAV for in vivo expression of Sesn1 and Sesn2 were generated and provided by the Virus Production Unit (UPV, UAB, Barcelona). AAVs were diluted in 0.9% NaCl at 0.25*10^13^ gc/ml and directly injected into muscles (40 µl/TA and 10 µl/EDL and soleus muscles). AAV-GFP was used as a control. Four days after transduction mice were subjected to the immobilization protocol.

### Pharmacological treatments

Mice were injected with rapamycin (4 mg/kg body weight) (which induces autophagy) or vehicle (DMSO) intraperitoneally (i.p.) every other day for 2 weeks. Colchicine (which inhibits autophagy) was injected i.p. (0.4 mg/kg*day) 2 days before sacrifice. The proteasome inhibitor Bortezomib (0.1 mg/kg) or vehicle (DMSO) was injected i.p. every other day for 2 weeks. Mice were treated with 3 mM spermidine in drinking water for 2 weeks.

### Muscle force measurement

Ex vivo force measurements of EDL and soleus muscles was assessed as previously described^41^. Briefly, mice were sacrificed, and muscles were immediately excised and placed into a dish containing oxygenated Krebs–Henseleit solution. Muscles were mounted vertically in a temperature controlled (30 °C) chamber and immersed in the Krebs–Ringer bicarbonate buffer solution, with 10 mM glucose, also continuously oxygenated. One end of the muscle was linked to a fixed clamp, while the other end was connected to the lever-arm of an Aurora Scientific Instruments 300B actuator/transducer system, using a nylon thread. The optimum muscle length (Lo) was determined from micromanipulations of muscle length to produce the maximum isometric twitch force. Maximum isometric-specific tetanic force was determined from the plateau of the curve of the relationship between specific isometric force with a stimulation frequency ranging from 1 to 200 Hz. Force was normalized per muscle area (determined by dividing the muscle mass by the product of longitude and the density of muscle (1.06 mg/mm^3^)) to calculate the specific force (mN/mm^2^).

### Muscle histology and immunohistochemistry

Muscles were embedded in OCT solution (TissueTek), frozen in isopentane cooled with liquid nitrogen and stored at −80 °C until analysis. 10 μm muscle cryosections were collected and stained for hematoxylin/eosin (H/E) or Sirius red (Sigma-Aldrich).

For immunohistochemistry assays, muscle cryosections were examined by standard immunohistochemical procedures for the expression of myosin heavy chain (MHC) isoforms. The primary monoclonal antibodies employed were anti-myosin I (A4.840), anti-myosin IIA (A4.74), and anti-myosin IIB (BF-F3) (Developmental Studies Hybridoma Bank).

Digital images were acquired using the Leica DMR600B microscope equipped with a DFC300FX camera. Fiber type distribution, CSA, and percentage of muscle area positive for Sirius red staining were quantified using Image J software, as previously reported^42,43^.

For myonuclei quantification, muscle sections were immunostained for dystrophin (1/400), the secondary antibody was coupled to Alexa-488 and nuclei were stained with DAPI (Invitrogen). Images were acquired using a Leica TCS SP5 confocal scanning microscope system.

### Fluorescence microscopy analysis of muscle sections

TA muscles were removed, fixed in PFA 2% for 4 h at 4 °C and incubated with 15% sucrose overnight at 4 °C. Then, muscles were embedded in OCT solution (TissueTek), immediately frozen in liquid nitrogen-cooled isopentane and stored at −80 °C. 10 μm cryosections of TA muscle (which has been transfected with mRFP-GFP-LC3^44^) were analyzed using a Leica TCS SP5 confocal scanning microscope system. Colocalization of RFP-LC3 and GFP-LC3 puncta was determined on the maximum projection of 10-z sections. Note: Measuring autophagy flux through this method is based on the concept of lysosomal quenching of GFP. GFP is a stably folded protein and relatively resistant to lysosomal proteases. However, the low pH inside the lysosome quenches the fluorescent signal of GFP, which makes it difficult to trace the delivery of GFP–LC3 to lysosomes. In contrast, RFP exhibits more stable fluorescence in acidic compartments, and mRFP–LC3 can be readily detected in autolysosomes. By exploiting the difference in the nature of these two fluorescent proteins (that is, lysosomal quenching of GFP fluorescence versus lysosomal stability of RFP fluorescence), autophagic flux can be morphologically traced with an mRFP–GFP–LC3 tandem construct. With this tandem construct, autophagosomes and autolysosomes are labeled with yellow (mRFP and GFP) and red (mRFP only) signals, respectively.

### RNA isolation, reverse transcription, and quantitative PCR

Total RNA from TA muscles was isolated with QIAzol Lysis Reagent (Qiagen) and quantified with Nanodrop. M-MLV Reverse Transcriptase (Promega) was used to synthesize cDNAs from 1 µg total RNA following the manufacturer’s instructions. RT-qPCR reactions were performed with SYBR Green in 384-well plates using the Roche LC-480 cycler (Roche Applied Science). All data were normalized to L7 expression. Primer sequences are listed in Table 1.

**Table 1:** Primers used for qPCR.

Gene	Species	Forward primer	Reverse primer
*Sesn1*	Mouse	GTCTGGATAACATCACATTAG	CCAGGTAGGAACACTGATGC
	Human	CAGCATTGGAAAACATTAGGCAA	CCGAAGACTCGGTATTTGAAAGC
*Sesn2*	Mouse	TAGCCTGCAGCCTCACCTAT	TATCTGATGCCAAAGACGCA
*Sesn3*	Mouse	CATGCGTTTCCTCACTCAGA	GGCAAAGTCTTCGTACCCAA
*Fbxo32*	Mouse	AAGGCTGTTGGAGCTGATAGCA	CACCCACATGTTAATGTTGCCC
*Trim63*	Mouse	TGCCTGGAGATGTTTACCAAGC	AAACGACCTCCAGACATGGACA
*Ctsl*	Mouse	GTGGACTGTTCTCACGCTCAAG	TCCGTCCTTCGCTTCATAGG
*FoxO1*	Mouse	CTGGGTGTCAGGCTAAGAGT	GGGGTGAAGGGCATCTTT
*FoxO3*	Mouse	CTGGGGGAACCTGTCCTATG	TCATTCTGAACGCGCATGAAG
*FoxO4*	Mouse	CTTCCTCGACCAGACCTCG	ACAGGATCGGTTCGGAGTGT
*Atg7*	Mouse	TCTGGGAAGCCATAAAGTCAGG	GCGAAGGTCAGGAGCAGAA
*L7*	Mouse	GAAGCTCATCTATGAGAAGGC	AAGACGAAGGAGCTGCAGAAC

### Transcriptomic analysis

For RNAseq analysis, total RNA from TA muscles was extracted using a protocol combining QIAzol Lysis Reagent and RNAeasy minikit columns (Qiagen) following the manufacturer’s instructions. RNAseq services were provided by the CNIC Genomics Unit, including quality control tests of total RNA using Agilent Bioanalyzer and Nanodrop spectrophotometry. cDNA library preparation and amplification were performed from 200 ng total RNA using NEBNext Ultra RNA Library Prep Kit for Illumina. RNAseq analysis was performed with 3–4 samples per condition, using Illumina Hiseq 2500. Sequencing reads were pre-processed by means of a pipeline that used FastQC, to asses read quality, and Cutadapt 1.7.1 to trim sequencing reads, eliminating Illumina adaptor remains, and to discard reads that were shorter than 30 bp. The resulting reads were mapped against the mouse transcriptome (GRCm38, release 76; aug2014 archive) and quantified using RSEM v1.2.20. Data were then processed with a differential expression analysis pipeline that used Bioconductor package LIMMA for normalization and differential expression testing. For differential expression analysis we filtered for genes that showed at least Log2FC 0.25 (≥+0.25 for upregulation; and ≤−0.25 for downregulation) and an adj. *p* value < 0.05.

### FoxO promoter-reporter assay

TA muscles from Sesn1^WT^ and Sesn1^SkM-Tg^ mice were electrotransferred with a reporter plasmid with three copies of forkhead responsive element linked to the luciferase reporter gene (FHRE-luciferase; Addgene). After 3 days of immobilization, muscles were collected and luciferase activity was measured in muscle homogenates by using Dual-Luciferase Reporter Assay Kit (Promega Corporation, USA). Values were normalized to non-immobilized muscles.

### Muscle protein extraction and Western blotting

Total homogenates from skeletal muscle were obtained in IP buffer (50 mM Tris–HCl pH 7.5, 150 mM NaCl, 1% NP-40, 5 mM EGTA, 5 mM EDTA, 20 mM NaF, 25 mM β-glycerophosphate, 0.1 mM sodium vanadate, 1 mM PMSF) supplemented with protease and phosphatase inhibitors (Complete Mini, Roche Diagnostic Corporation; phosphatase inhibitor cocktail, Sigma). Protein concentration was measured using the Bradford method (Protein Assay, Bio-Rad). 40 µg of protein were resolved by SDS–PAGE and transferred to PVDF membranes (Millipore). Membranes were blocked with 5% milk in TBS-T for 1 h and incubated with primary antibodies overnight at 4 °C in 5% BSA in TBS-T (Tubulin 1:4000 and others 1:1000). Proteins were detected by the ECL method and quantified by scanning densitometry. The antibodies used are listed in Table 2. Uncropped versions of all blots shown in figures are supplied in the Source Data File.

**Table 2:** Antibodies.

Antibody	Source	Reference
Rabbit anti-Sesn1	Provided by Dr. Jun Hee Lee	
Rabbit anti-Sesn2	Proteintech	Cat#10795-1-AP
Rabbit anti-phospho-AKT (Ser473)	Cell Signaling Technology	Cat#9271
Rabbit anti-phospho-AKT (Trh308)	Cell Signaling Technology	Cat#4056
Mouse anti-AKT (pan)	Cell Signaling Technology	Cat#2920
Rabbit anti-phospho-FoxO1 (Thr24)/FoxO3a (Thr32)	Cell Signaling Technology	Cat#9464
Rabbit anti-phospho-S6 (Ser235/236)	Cell Signaling Technology	Cat#4858
Mouse anti-S6	Cell Signaling Technology	Cat#2317
Anti-Phospho-AMPKα (Thr172)	Cell Signaling Technology	Cat#2531
Anti-AMPKα	Cell Signaling Technology	Cat#2532
Rabbit anti-phospho-ULK1	Cell Signaling Technology	Cat#14202
Rabbit anti-ULK1	Cell Signaling Technology	Cat#8054
Rabbit anti-LC3B	Cell Signaling Technology	Cat#2775
Mouse anti-Tubulin	Sigma Aldrich	Cat#T-6199
Mouse anti-GAPDH	Santa Cruz Biotechnology	Cat#sc-32233

### Proteasome activity in muscle

Proteasome activity in total homogenates from TA muscles was determined by evaluating the cleavage of specific fluorogenic substrates. Muscles were homogenized in lysis buffer (50 mM Tris–HCl pH 7.5, 250 mM Sucrose, 5 mM MgCl_2_, 0.5 mM EDTA, 2 mM ATP, and 1 mM DTT) and centrifuged at 12,000 × *g* for 30 min at 4 °C. The supernatant was collected and protein concentration determined by the method of Bradford. For chymotrypsin-like activity, aliquots of 20 μg protein were incubated for 60 min at 37 °C in the presence of 100 μM of the fluorogenic substrate succinyl-Leu-Leu-Val-Tyr-7-amino-4-methylcoumarin (Suc LLVY-AMC). Each assay was conducted in the absence and presence of the specific proteasomal inhibitor MG132 (Sigma-Aldrich) at 20 μM. Fluorescence was read with a spectrofluorometer (390 nm excitation/460 nm emission; Tecan Infinite M200). The activity was expressed as units of fluorescence per microgram of protein, as a percentage of the control group. All samples were assayed in triplicate using at least four animals.

### Bioinformatic analysis

Hierarchical clustering of expression values (after filtering expression values below one in all the samples) was carried out with Morpheus (https://software.broadinstitute.org/morpheus/) using one minus Pearson correlation with a complete linkage. GSEA of Sestrin-regulated genes was performed using GSEA web interface with the Molecular Signatures Database “hallmarks” and “transcription factor binding targets” genesets, to reveal pathways and cis-regulatory motifs which can function as potential transcription factor-binding sites, respectively^45^. The Java implementation code from the Broad Institute was used for direct GSEA comparisons of the raw data from our RNA-seq experiments or with GEO: GSE5395 data set with the “AKT-MTOR signaling” gene set (generated by combining the “MTORC1 Signaling” and the “PI3K-AKT- MTOR signaling” Molecular Signatures Database hallmarks), the growth and atrophy regulators gene set (assembled by combining the following Gene Ontology categories: positive and negative regulations of insulin-like growth factor receptor signaling pathway, positive and negative regulations of TORC1 signaling and positive and negative regulations of muscle atrophy) and the atrogenes gene set (that was manually curated from selected bibliography^1–4,6,7,22,24^). Bubble plots were generated using ggplot2 library in R or Seaborn in Python. Venn diagrams were generated using The BEG Ugent tool (http://bioinformatics.psb.ugent.be/webtools/Venn/).

### Statistical analysis

For mouse experiments, no specific blinding method was used, but mice in each sample group were selected randomly. The sample size (*n*) of each experimental group is described in each corresponding figure legend.

GraphPad Prism software was used for all statistical analyses. Quantitative data displayed as histograms are expressed as means ± standard error of the mean (represented as error bars). Results from each group were averaged and used to calculate descriptive statistics.

Unpaired *t*-test (independent samples, two-sided) was used for pairwise comparisons among groups at each time point, unless indicated in figure legends. Statistical significance was set at a *p* < 0.05.

## Supplementary information

Supplementary Information

Supplementary Data 1

Supplementary Data 2

Supplementary Data 3

Description of Additional Supplementary Files

## Data Availability

The data that support the findings of this study are available from the corresponding author upon reasonable request. Source data used to generate Figs. 1–5 and Supplementary Figs. 1–7 are provided in the Source Data file. The RNA-sequencing data have been deposited in the Gene Expression Omnibus (GEO) database under accession code: GSE136866.

## Source Data

Source Data File
