# Supplementary material for: Sestrin prevents atrophy of disused and aging muscles by integrating anabolic and catabolic signals
Source: Nat Commun. 2020 Jan 13;11:189. doi: 10.1038/s41467-019-13832-9 (PMC6955241; doi:10.1038/s41467-019-13832-9)
Supplement: Supplementary file 1 — Supplementary Information [file 41467_2019_13832_MOESM1_ESM.pdf]

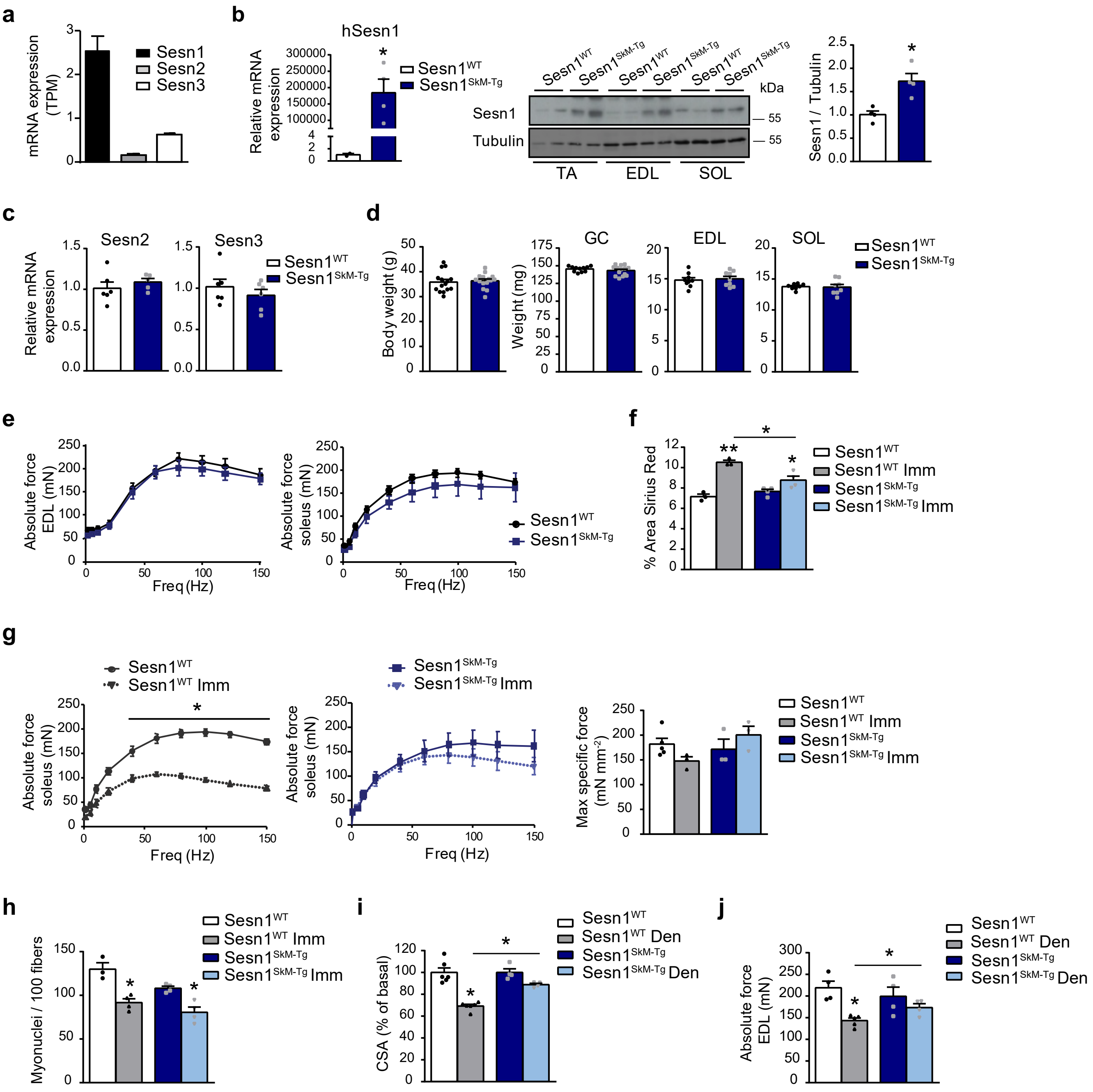

Supplementary Figure 1. Functional characterization of muscles from Sesn1^{SkM-Tg} mice

a, RNAseq-determined Sesn1-3 mRNA expression levels in skeletal muscle of young mice. **b,** qPCR (left) and Western blot analysis and its quantification (right) of human Sesn1 mRNA and protein expression respectively in skeletal muscle from Sesn1^{WT} and Sesn1^{SkM-Tg} young mice (4 months old). **c,** qPCR analysis of Sesn2 and Sesn3 expression in TA muscles of Sesn1^{WT} and Sesn1^{SkM-Tg} young mice. **d,** Body and muscle weight of young Sesn1^{WT} and Sesn1^{SkM-Tg} mice. **e,** Force-frequency curves of EDL and soleus muscles from Sesn1^{WT} and Sesn1^{SkM-Tg} mice in basal conditions. **f,** Quantification of Sirius Red staining of TA muscle sections from young Sesn1^{WT} and Sesn1^{SkM-Tg} mice in basal state and 10 days post-immobilization. **g,** Force measurements in soleus muscles of Sesn1^{WT} and Sesn1^{SkM-Tg} mice in basal conditions and after 10 days of limb immobilization. Charts show force-frequency curves (left) and maximum specific force (right). **h,** Myonuclei number in TA muscles from Sesn1^{WT} and Sesn1^{SkM-Tg} mice in basal conditions and after 10 days of immobilization. **i,** Mean fiber CSA of TA muscles from Sesn1^{WT} and Sesn1^{SkM-Tg} mice in basal conditions and at 10 days post denervation. Results are expressed as percentage of CSA in basal conditions. **j,** Force measurements in EDL muscles of Sesn1^{WT} and Sesn1^{SkM-Tg} mice in basal conditions and after 10 days of denervation. All data are shown as mean with SEM. Comparisons by paired (i) or unpaired (others) Student t-test (*$p<0.05$ and **$p<0.01$). TA, tibialis anterior; SOL, soleus and GC, gastrocnemius. N=5-7 mice per genotype for **e**, n=3-4 mice per group for **f** and **h**, n=3-7 animals per group for **g** and n=4-6 mice for **i-j**. Source data are provided as a Source Data file.

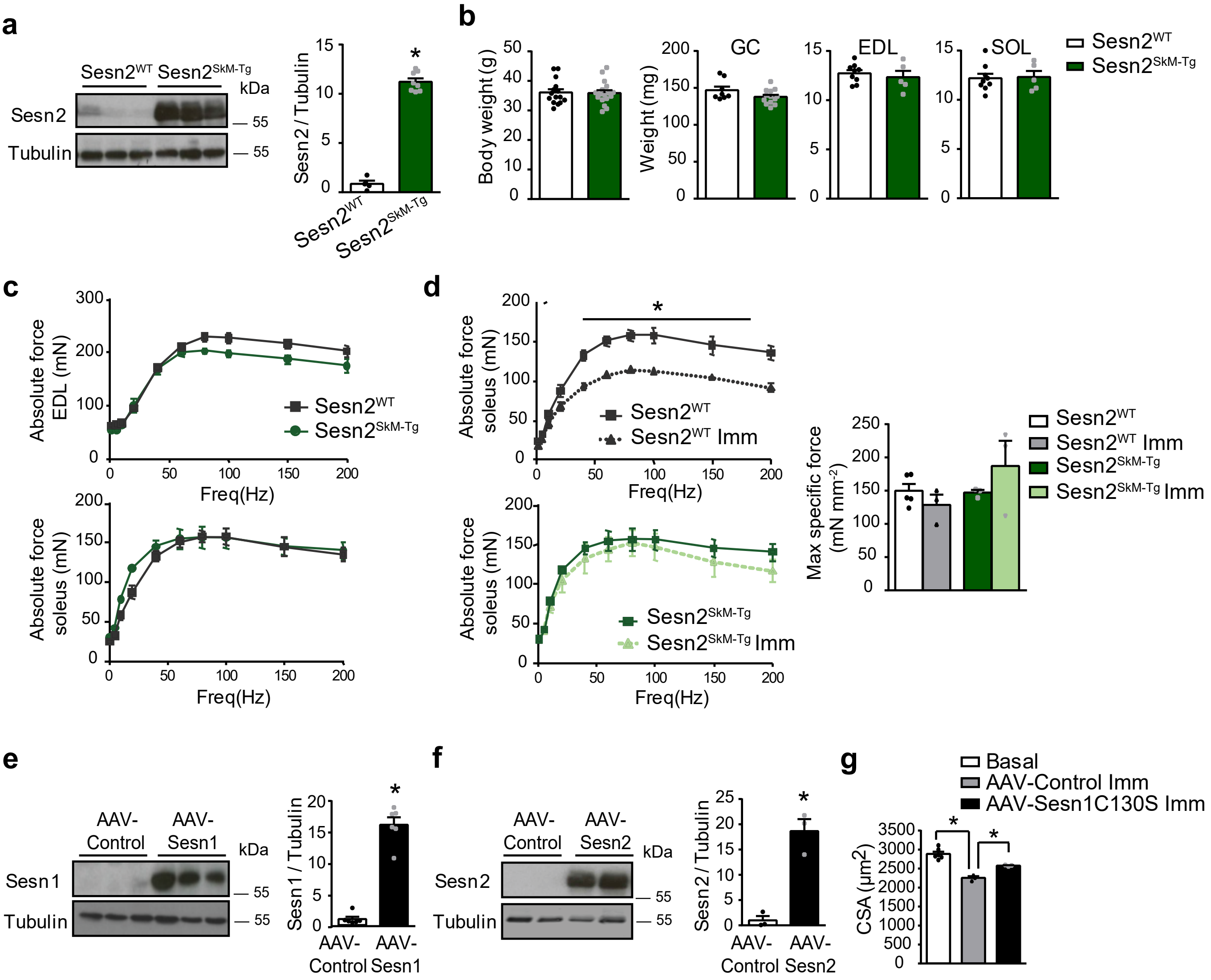

Supplementary Figure 2. Functional characterization of muscles from Sesn2$^{SkM\text{-}Tg}$ mice

a, Western blot and quantification of Sesn2 protein levels in skeletal muscle from Sesn2^{WT} and Sesn2$^{SkM\text{-}Tg}$ mice. **b,** Body and muscle weight of young Sesn2^{WT} and Sesn2$^{SkM\text{-}Tg}$ mice (4 months old). **c,** Force-frequency curves of EDL and soleus muscles from Sesn2^{WT} and Sesn2$^{SkM\text{-}Tg}$ mice in basal conditions. **d,** Force measurements in soleus muscles of Sesn2^{WT} and Sesn2$^{SkM\text{-}Tg}$ mice in basal conditions and after 10 days of limb immobilization. Charts show force-frequency curves and maximum specific force. **e, f,** Western blot analysis and quantification of Sesn1 and Sesn2 expression in TA muscles transduced with AAV-Sesn1 (**e**) or AAV-Sesn2 (**f**). **g,** Mean myofiber CSA in TA muscles transduced with AAV-Sesn1C130S or AAV-Control and immobilized for 10 days. All data are shown as mean with SEM. Comparisons by unpaired Student t-test (*$p<0.05$). Sample numbers were n=3-5 mice per group for **c-d**, n=3 animals for **g**. Source data are provided as a Source Data file.

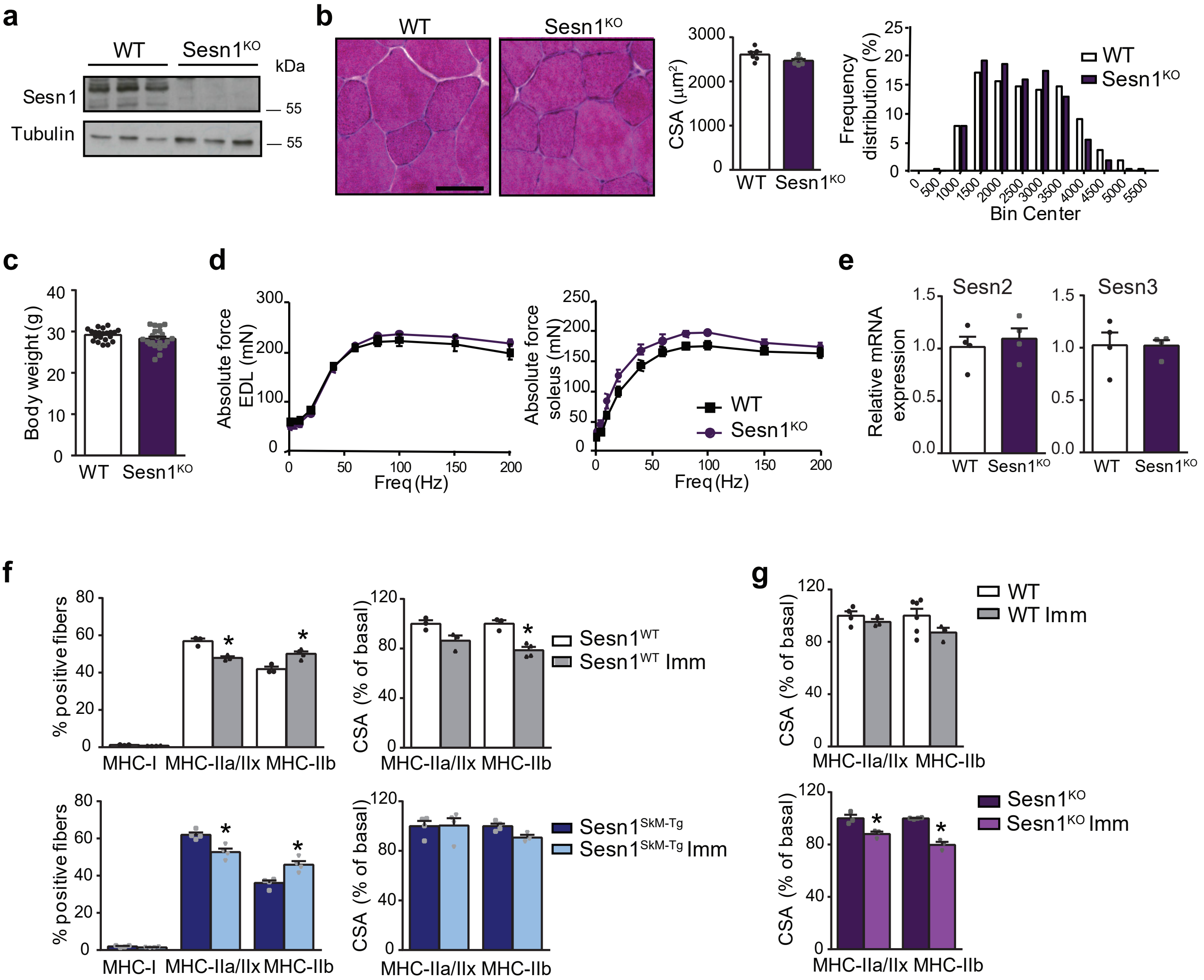

Supplementary Figure 3. Functional characterization of muscles from Sesn1^{KO} mice

a, Representative Western blot showing Sesn1 protein levels in WT and Sesn1^{KO} mice. **b,** Representative H/E staining pictures of TA muscle sections from WT and Sesn1^{KO} mice (left), quantification of mean myofiber CSA (center) and myofiber-size frequency distribution (right panel). Scale bar=50 µm. **c,** Body weight of young WT and Sesn1^{KO} mice. **d,** Force-frequency curves of EDL and soleus muscles from WT and Sesn1^{KO} mice in basal conditions. **e,** qPCR analysis of Sesn2 and Sesn3 mRNA expression in skeletal muscle from WT and Sesn1^{KO} mice. **f,** Percentage of type I, IIA/IIX, and IIB fibers, based on myosin heavy chain (MHC) expression, in TA muscle sections from Sesn1^{WT} (top) and Sesn1$^{SkM\text{-}Tg}$ mice (bottom) in basal conditions and after 10 days of immobilization (left). CSA of type IIA/IIX and IIB fibers in basal conditions and after 10 days of immobilization (right). **g,** Mean CSA of type IIA/IIX and IIB fibers in TA muscles from WT and Sesn1^{KO} mice in basal conditions and after 10 days of immobilization. All data are shown as mean with SEM. Comparisons by unpaired Student t-test (*$p<0.05$). Sample numbers were n=5-7 mice for **b-d** and n=3-5 mice per condition for **f-g**. Source data are provided as a Source Data file.

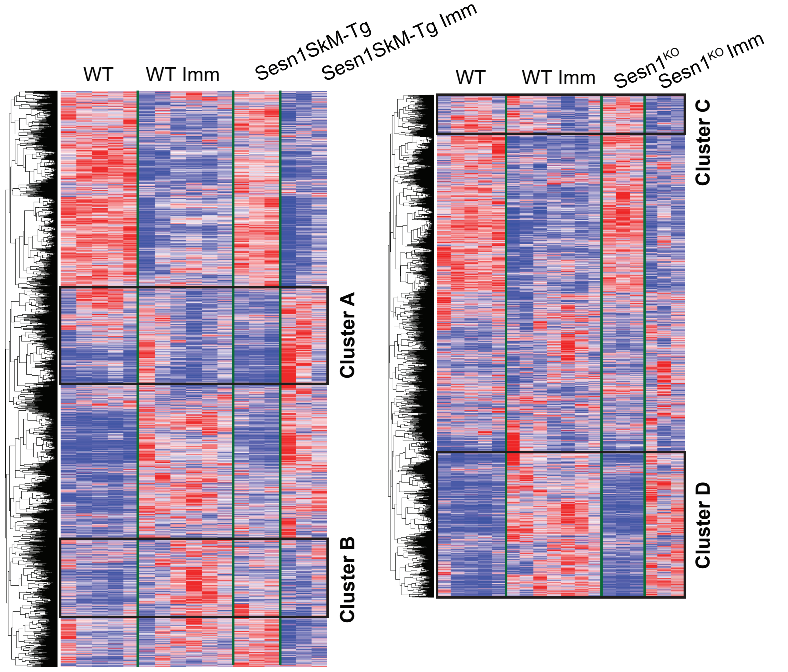

Sestrin-regulated genes
Common genes between clusters A and C, and clusters B and D = **2065 genes**

GSEA canonical pathways	Genes	p-value
Immune system	146	6.22E-45
Adaptive immune system	96	1.91E-34
HIV infection	54	2.33E-28
Class I MHC mediated antigen processing presentation	57	1.71E-26
**Antigen processing ubiquitination proteasome degradation**	51	4.36E-25
Apoptosis	42	6.16E-24
Downstream signaling events of cell receptor Bcr	33	6.97E-22
Regulation of apoptosis	26	3.78E-21
**Proteasome**	24	4.68E-21
Activation of NFκB in B cells	27	4.76E-21
Signaling by the B cell receptor Bcr	36	7.81E-21
Signaling by Wnt	27	7.83E-21
Regulation of ornithine decarboxylase Odc	24	8.82E-21
**Autodegradation of the E3 ubiquitin ligase Cop1**	24	2.96E-20
**Scf beta Trcp mediated degradation of Emi1**	24	2.96E-20

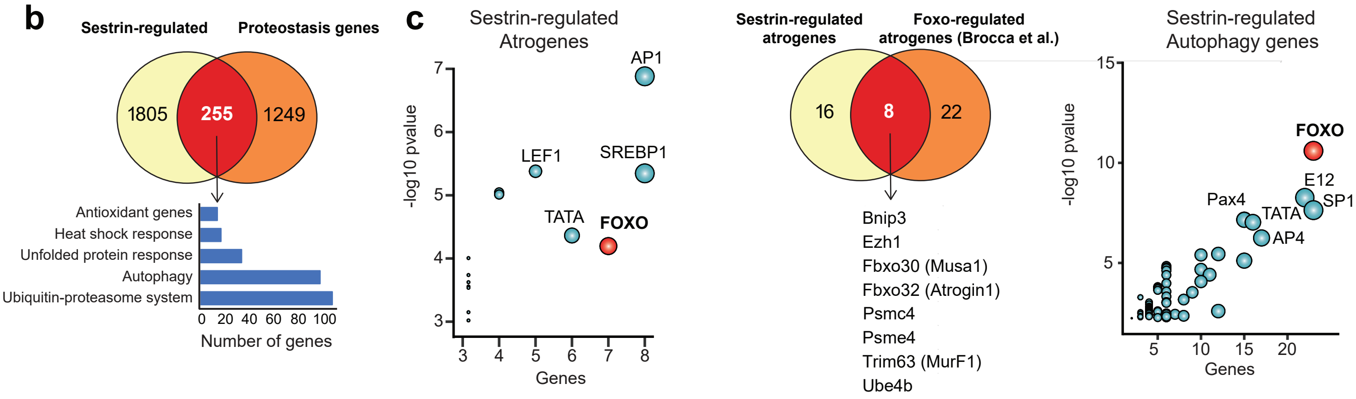

Supplementary Figure 4. Sestrin regulates the expression of critical atrogenes in disused muscles
a, Unsupervised hierarchical clustering heat map of normalized gene expression in 3-day immobilized TA muscles compared with basal conditions in the indicated mouse genotypes. Each column corresponds to an individual sample. Selected clusters are shown. Cluster A/B shows genes downregulated/upregulated in immobilized WT muscles compared to basal, which behave differently in Sesn-expressing muscles, and Cluster C/D to genes showing decreased/increased expression in Sesn1-deficient mice. Addition of the common genes between clusters A and C, and between cluster B and D defined the "sestrin-regulated gene set". The table shows GSEA-identified canonical pathways enriched in the sestrin-regulated gene set. **b,** Venn diagram showing genes common to the sestrin-regulated gene set (defined in a) and a full set of genes implicated in proteostasis pathways[32]. The bar chart shows sestrin-regulated proteostasis genes subdivided into individual pathways. **c,** Bubble plot of enriched transcription factor binding sites (GSEA) among autophagy genes (right) and atrogenes (left) belonging to the sestrin-regulated gene set defined in a. Comparison of FoxO-regulated atrogenes from Brocca et al.[24] and the list of sestrin regulated atrogenes (middle).

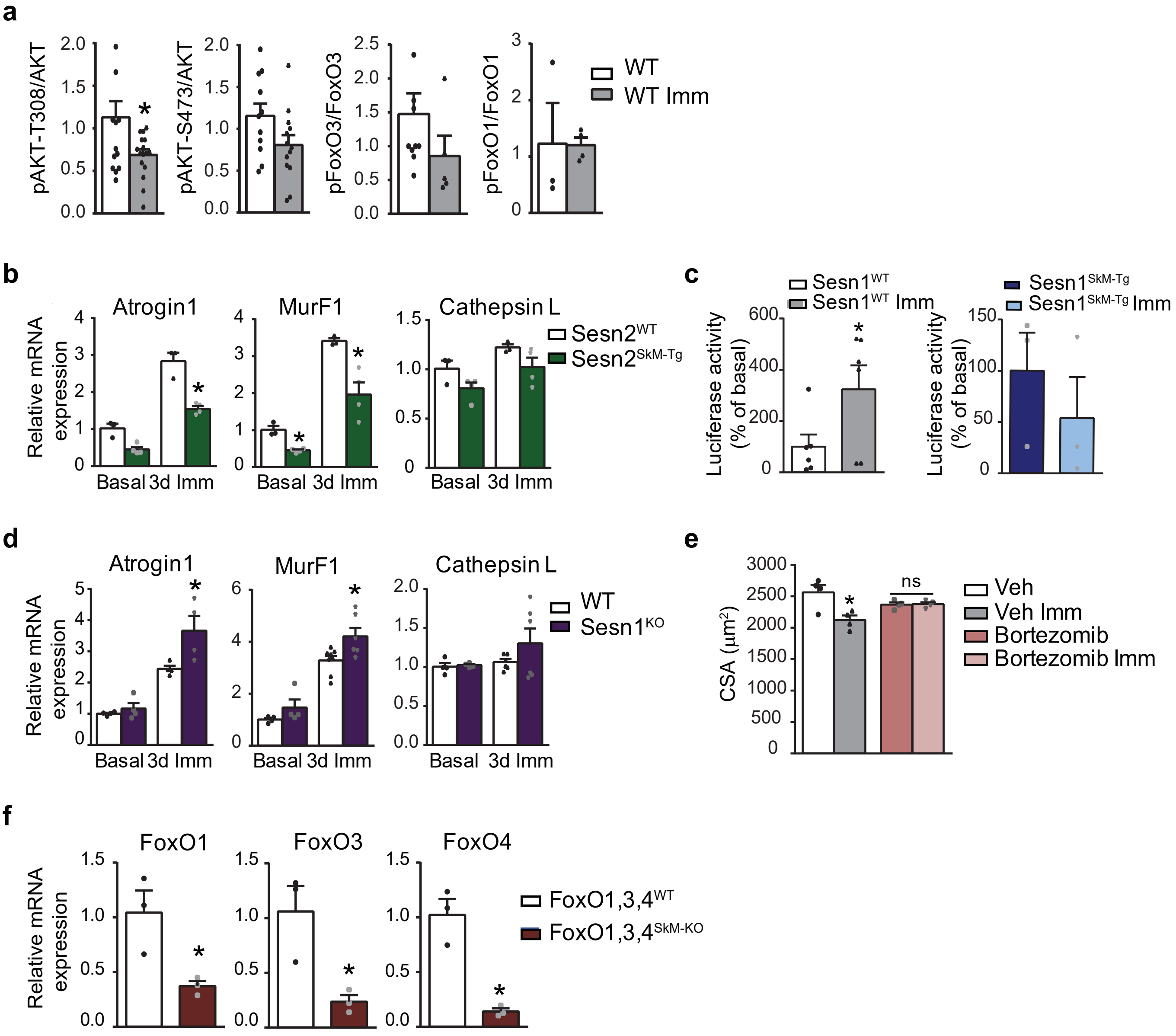

Supplementary Figure 5. Sestrin regulates the expression of proteasome-related genes in atrophying muscles
a, Quantification of the Western blot analyses of FoxO1, FoxO3 and AKT phosphorylation levels in muscles of WT mice in basal conditions and after 3 days of immobilization. **b,** Atrogin1, MurF1, and cathepsin L mRNA levels in muscles from Sesn2^{WT} and Sesn2$^{SkM\text{-}Tg}$ mice in basal conditions and 3 days post-immobilization. **c,** Luciferase activity in TA muscle homogenates from Sesn1^{WT} and Sesn1$^{SkM\text{-}Tg}$ mice electrotransferred with the FHRE-luciferase reporter plasmid and immobilized for 3 days. **d,** Atrogin1, MurF1, and cathepsin L mRNA levels in muscles from WT and Sesn1^{KO} mice in basal conditions and 3 days post-immobilization. **e,** Mean fiber CSA of TA muscles from WT mice treated with vehicle or the proteasome inhibitor bortezomib in basal conditions and at 10 days post-immobilization. **f,** mRNA expression levels of FoxO1,3,4 in skeletal muscle from FoxO1,3,4^{WT} and FoxO1,3,4$^{SkM\text{-}KO}$ mice. All data are shown as mean with SEM. Comparisons by paired (**c**) or unpaired (others) Student t-test (*p<0.05). Sample numbers were n=3-7 mice per group for **b** and **d**, n=3-6 mice for **c**, n=4 mice per condition for **e** and n=3 mice for **i.**

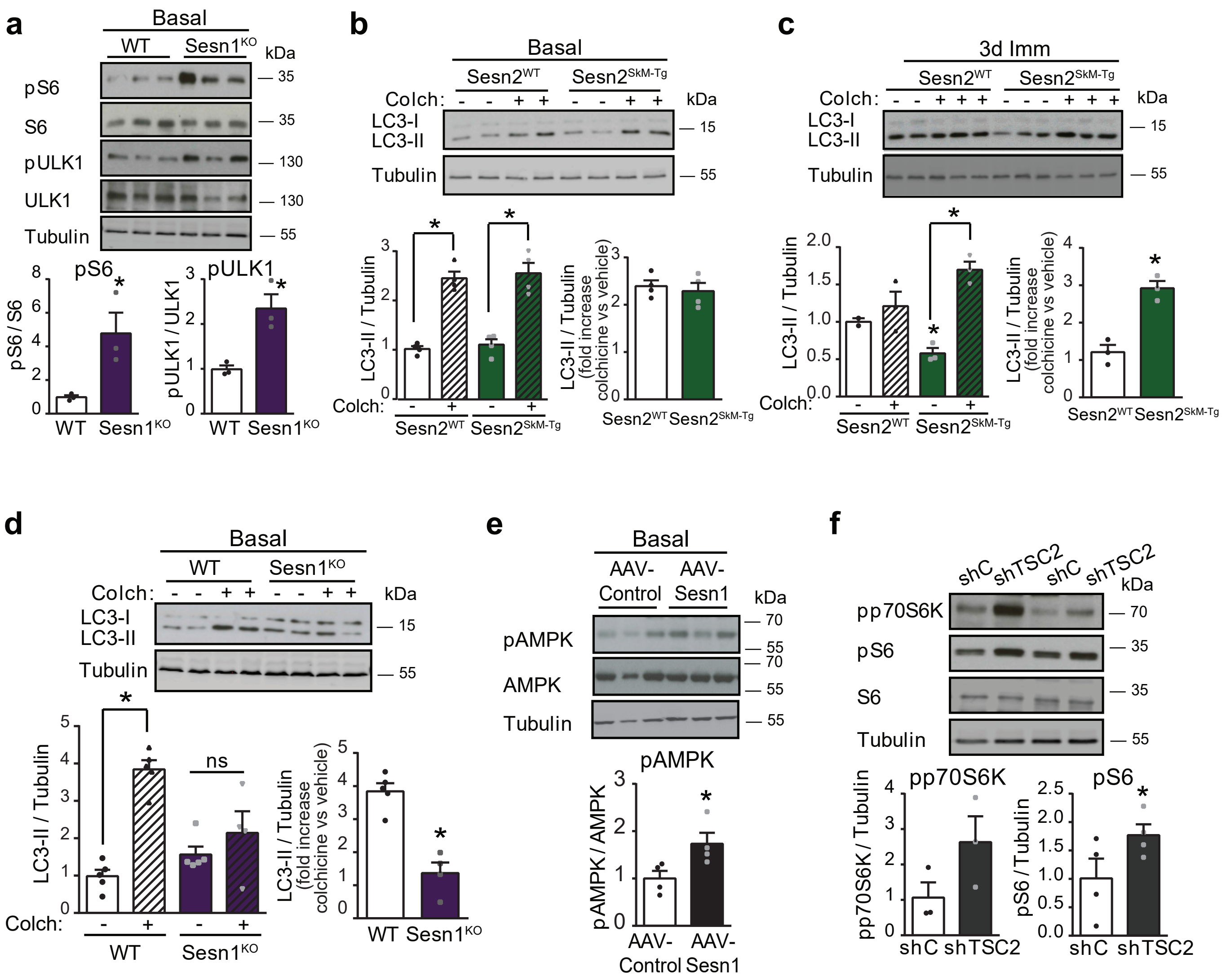

Supplementary Figure 6. Sestrins inhibit mTORC1 and maintain autophagy in disused muscles
a, Western blot analysis of S6 and ULK1 phosphorylation in TA muscles from WT and Sesn1^{KO} mice in basal conditions. **b** and **c,** Western blot analysis and quantification of LC3I and LC3II content in TA homogenates from Sesn2^{WT} and Sesn2$^{SkM\text{-}Tg}$ mice in basal conditions (**b**) and after 3 days of immobilization (**c**). The right chart shows the fold increase in LC3II content in colchicine (Colch) treated versus vehicle-treated mice (autophagic flux). **d,** Western blot analysis of LC3I and LC3II in homogenates of WT and Sesn1^{KO} TA muscles in basal conditions. **e,** Western blot analysis of AMPK phosphorylation in muscles transduced with AAV-Sesn1 or AAV-Control in basal conditions. **f,** Western blot analysis of mTORC1 signaling activation in TA muscles electrotransferred with control short hairpin (sh) and sh against TSC2 (shTSC2); blots were probed for phosphorylated p70S6K and S6 and total S6. All data are shown as mean with SEM. Comparisons by paired unpaired Student t-test (*p<0.05). For **b-d** values were normalized to control vehicle conditions. Sample numbers were n=3-4 animals per condition for **a-c** and **f,** n=4 mice for **e** and n=5 mice for **d**.

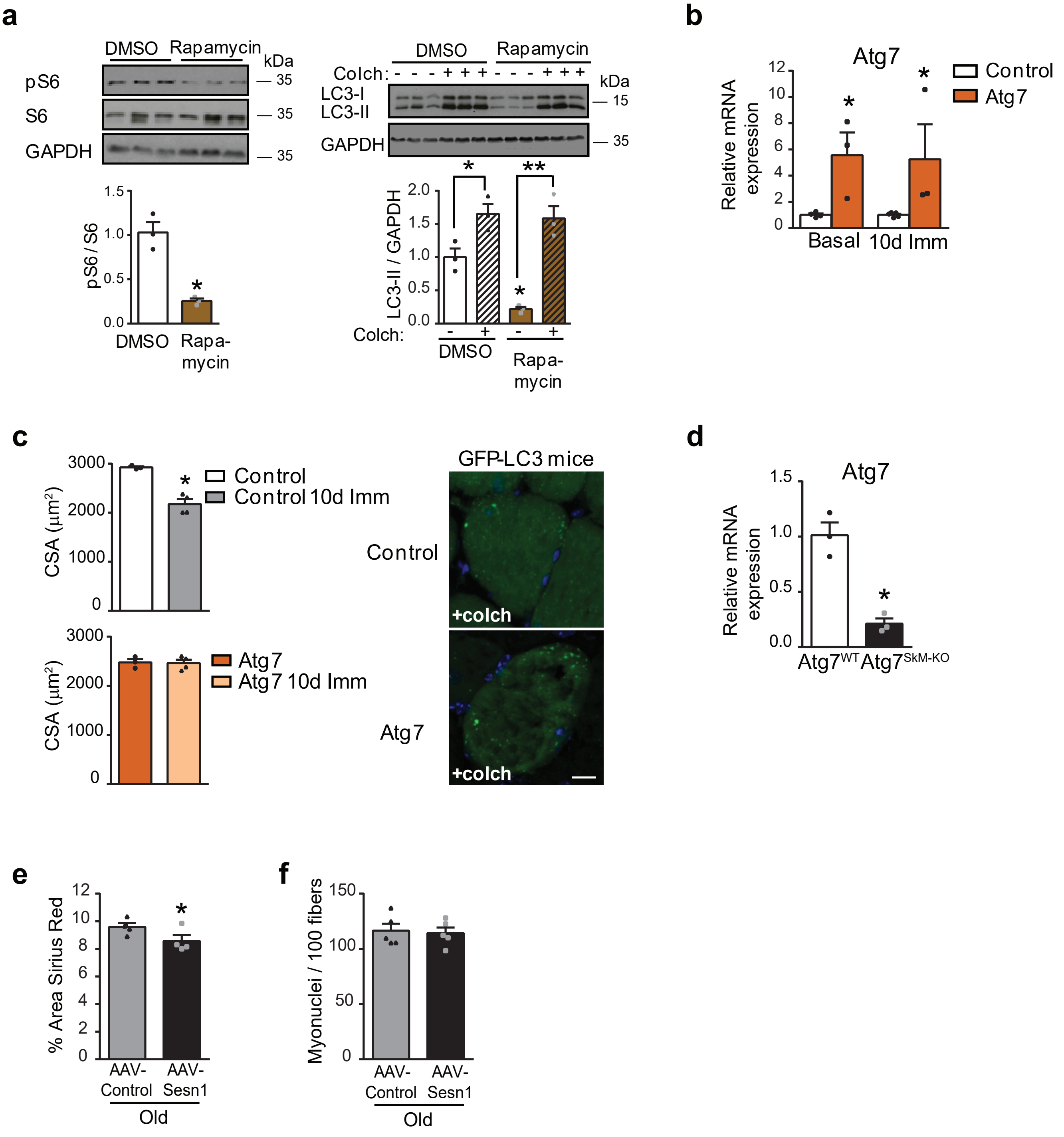

Supplementary Figure 7. Autophagy induction prevents disuse-induced muscle atrophy
a, Western blot analysis of mTORC1 inhibition and autophagy induction in WT mice treated with rapamycin or vehicle (DMSO); blots were probed for phosphorylated and total S6 and LC3. **b,** qPCR analysis of Atg7 overexpression in WT TA muscles electrotransferred with a plasmid encoding Atg7 and then immobilized for 10 days. **c,** Mean myofiber CSA of fibers from TA muscles of WT mice treated as in **h**. Right panels show representative confocal images of TA muscle from LC3-GFP mice electrotransferred with a plasmid encoding Atg7 and treated with colchicine to assess autophagosome accumulation. Scale bar=10 µm. **d,** mRNA expression levels of Atg7 in skeletal muscle from $Atg7^{WT}$ and $Atg7^{SkM\text{-}KO}$ mice. **e,** Quantification of Sirius Red staining of TA muscle sections from 24 month-old WT mice transduced with AAV-Sesn1 for 28 days. **f,** Number of myonuclei in TA muscle of old mice transduced with AAV-Sesn1 for 28 days. All data are shown as mean with SEM. Comparisons by paired (**e**) or unpaired (others) Student t-test (*$p<0.05$). Sample numbers were n=3-4 animals per condition for **a-d**, n=4 mice for **e** and n=5 mice for **f**.
