# Supplementary material for: Sestrin prevents atrophy of disused and aging muscles by integrating anabolic and catabolic signals
Source: Nat Commun. 2020 Jan 13;11:189. doi: 10.1038/s41467-019-13832-9 (PMC6955241; doi:10.1038/s41467-019-13832-9)
Supplement: Supplementary file 5 — Description of Additional Supplementary Files [file 41467_2019_13832_MOESM5_ESM.docx]

**Title:** Supplementary Data 1
**Description:** List of dysregulated genes in different models of muscle atrophy.

**Title:** Supplementary Data 2
**Description:** Enriched GSEA Hallmarks in the dysregulated genes upon 3-day muscle immobilization in all mice genotypes.

**Title:** Supplementary Data 3
**Description:** Hierarchical clustering analysis to identify genes regulated by both immobilization and sestrins. Selected clusters and enriched canonical pathways are shown.
